# Risk of Self-harm in Children and Adults With Autism Spectrum Disorder: A Systematic Review and Meta-analysis

**DOI:** 10.1001/jamanetworkopen.2021.30272

**Published:** 2021-10-19

**Authors:** Ashley Blanchard, Stanford Chihuri, Carolyn G. DiGuiseppi, Guohua Li

**Affiliations:** 1 Department of Emergency Medicine, Columbia University Vagelos College of Physicians and Surgeons, New York, New York; 2 Department of Anesthesiology, Columbia University College of Physicians and Surgeons, New York, New York; 3 Department of Epidemiology, Colorado School of Public Health, University of Colorado Anschutz Medical Campus, Aurora; 4 Department of Epidemiology, Columbia University Mailman School of Public Health, New York, New York

## Abstract

**Importance:**

Multiple studies have reported that people with autism spectrum disorder (ASD) are at a higher risk for self-injurious behavior and suicide. However, the magnitude of this association varies between studies.

**Objective:**

To appraise the available epidemiologic studies on the risk of self-injurious behavior and suicidality among children and adults with ASD.

**Data Sources:**

PubMed, Embase, CINAHL, PsycINFO, and Web of Science were systematically searched for epidemiologic studies on the association between ASD and self-injurious behavior and suicidality. Databases were searched from year of inception to April through June 2020. No language, age, or date restrictions were applied.

**Study Selection:**

This systematic review and meta-analysis included studies with an observational design and compared self-injurious behavior (defined as nonaccidental behavior resulting in self-inflicted physical injury but without intent of suicide or sexual arousal) and/or suicidality (defined as suicidal ideation, suicide attempt, or suicide) in children (aged <20 years) or adults (aged ≥20 years) with ASD.

**Data Extraction and Synthesis:**

Information on study design, study population, ASD and self-harm definitions, and outcomes were extracted by independent investigators. Study quality was assessed using the Newcastle-Ottawa Scale. Overall summary odds ratios (ORs) and 95% CIs were estimated using DerSimonian-Laird random-effects models.

**Main Outcomes and Measures:**

The ORs for the associations of ASD with self-injurious behavior and suicidality were calculated. Analyses were stratified by study setting and age groups as planned a priori.

**Results:**

The search identified 31 eligible studies, which were of moderate to high quality. Of these studies, 16 (52%) were conducted in children, 13 (42%) in adults, and 2 (6%) in both children and adults. Seventeen studies assessed the association between ASD and self-injurious behavior and reported ORs that ranged from 1.21 to 18.76, resulting in a pooled OR of 3.18 (95% CI, 2.45-4.12). Sixteen studies assessed the association between ASD and suicidality and reported ORs that ranged from 0.86 to 11.10, resulting in a pooled OR of 3.32 (95% CI, 2.60-4.24). In stratified analyses, results were consistent between clinical and nonclinical settings and between children and adults.

**Conclusions and Relevance:**

This study found that ASD was associated with a substantial increase in odds of self-injurious behavior and suicidality in children and adults. Further research is needed to examine the role of primary care screenings, increased access to preventive mental health services, and lethal means counseling in reducing self-harm in this population.

## Key Points

**Question:**

What excess risk of self-harm, including self-injurious behaviors and suicidality, is associated with autism spectrum disorder (ASD)?

**Findings:**

This systematic review and meta-analysis of 31 studies found that the pooled odds of self-harm in people with ASD was more than 3 times the odds in people without ASD. The excess odds of self-harm were found in both children and adults (although a slightly higher risk was identified in adults) across geographic regions and regardless of study designs, methods, and settings.

**Meaning:**

Findings of this study suggest that children and adults with ASD are at a substantially increased risk for self-injurious behavior and suicidality.

## Teaser

This systematic review and meta-analysis reports the risk of self-harm, including self-injurious behaviors and suicidality, among pediatric and adult populations with autism spectrum disorder.

## Introduction

Autism spectrum disorder (ASD) is characterized by persistent deficits in social communication and interaction along with restricted, repetitive behaviors.^1^ In 2017, an estimated 5 437 988 US adults (2.21%) had ASD.^2^ Prevalence estimates in the US pediatric population have increased over the past several decades partly because of improved awareness, changes in documentation, and identification of milder cases without intellectual disability.^3^ In 2016, 1 in 59 children aged 8 years met surveillance criteria for ASD, and 1 in 40 children aged 3 to 17 years had a parent-reported ASD diagnosis.^4,5^

Among the myriad potential health problems for people with ASD is the excess risk of injury morbidity and mortality. Several epidemiologic studies using emergency department visit data have shown that children with ASD are at an elevated risk for injuries.^6,7,8,9,10^ Epidemiologic evidence has also indicated that people with ASD are at a heightened risk of injury mortality, with a risk of premature death that is 2- to 10-fold higher than in the general population.^11,12,13,14^ Self-harm may be a factor in this excess injury mortality given that people with ASD have a greater risk of self-injurious behavior, suicidal ideation, and suicide, although the estimated odds ratios (ORs) of self-harm in this population vary from 0.86 to 18.76.^15,16,17,18^

Several factors may explain the variability of existing estimates of self-harm risk in people with ASD, including common co-occurring mental health conditions that are associated with an increased risk of suicide.^19,20,21^ Pooled prevalence estimates demonstrate that 28% of people with ASD have co-occurring attention-deficit/hyperactivity disorder, 20% have co-occurring anxiety disorders, and 11% have co-occurring depressive disorders.^22^ These diagnoses are associated with a higher risk of suicide and increased prevalence of self-harm in this population. Estimates also vary among studies because of the outcome measures and comparison groups chosen, small sample sizes, and inclusion of clinical vs nonclinical samples.

Estimates may also vary depending on the definition of self-harm. Self-injurious behavior, such as hand hitting, self-cutting, or hair pulling, is common in the population with ASD, with an estimated prevalence of 42%.^23^ Self-injurious behavior is known to be associated with suicide, which has been documented in people with or without ASD.^12,24,25,26,27,28,29,30,31^ Among adolescents, nonsuicidal self-injury is associated with a markedly increased risk of suicide attempt (hazard ratio, 5.28; 95% CI, 1.80-15.47).^25^ This association is well established across the age spectrum.^32^ In a survey of people with ASD, Moseley et al^24^ found that every 1-point increase in score on the suicide item of the self-administered Suicide Behaviors Questionnaire was associated with a 2.2-fold increase in the risk of self-harm.

More precise estimates are needed to improve the recognition of and evidence-based interventions for self-harm in children and adults with ASD. In this systematic review with meta-analysis, we appraised the available epidemiologic studies on the risk of self-injurious behavior and suicidality among children and adults with ASD.

## Methods

We registered this systematic review and meta-analysis with PROSPERO (CRD42020175223) at the onset of the project; eMethods 1 in the Supplement describes deviations from the protocol. We followed the Preferred Reporting Items for Systematic Reviews and Meta-analyses (PRISMA) reporting guideline^33^ and the Meta-analysis of Observational Studies in Epidemiology (MOOSE) reporting guideline.

### Study Eligibility, Search Strategy, and Study Selection

Studies were eligible if they (1) had an observational design, such as cohort, case-cohort, case-control, and cross-sectional; (2) included the exposure group as ASD, which was ascertained by standardized tools, medical professionals, or *International Classification of Diseases* codes; (3) used an appropriate comparison group without ASD (eg, the general population or participants with non-ASD neurobehavioral disorder); (4) compared the prevalence or incidence of self-injurious behavior, suicidal ideation, suicide attempt, or suicide between those with ASD and those without ASD; and (5) presented quantitative data and at least 1 measure of association between ASD and self-injurious behavior or suicidality. Relevant literature was identified through a comprehensive search of PubMed (from 1996 to April 19, 2020), Embase (from 1980 to May 13, 2020), CINAHL (from 1982 to June 16, 2020), PsycINFO (from 1967 to June 16, 2020), and Web of Science (from 1900 to June 23, 2020). No language, age, or date restrictions were applied. The Boolean searches were completed with the assistance of a medical informationalist who specializes in systematic reviews and used relevant keywords and database-specific controlled vocabulary terms; the search strategies are available in eMethods 2 in the Supplement. Embase weekly search alerts were used from May 13, 2020, to January 31, 2021, to identify additional eligible studies. To identify unpublished or gray literature, we searched relevant conference abstracts, the National Institutes of Health RePORTER database, and the websites of organizations that are involved in ASD and self-injury research (eg, National Center on Birth Defects and Developmental Disabilities of the Centers for Disease Control and Prevention, The Child and Adolescent Health Measurement Initiative, and National Institute of Mental Health). We contacted authors of potentially eligible studies if data were missing or incomplete or if study populations seemed to be duplicates. No additional studies were obtained from author contacts or gray literature searches. Eligible studies spanned the entire age continuum; pediatric and adult studies were summarized separately.

All of the studies identified from searches were imported and deduplicated in Covidence systematic review software (Veritas Health Innovation). Two of us (A.B. and S.C.) screened study titles and abstracts for eligibility. Studies that we identified as potentially eligible were then reviewed in full text to ascertain their eligibility. Discrepancies in study selection were resolved by a discussion between the 2 of us or with a third author (G.L.). Reference lists and related article links of eligible studies were searched to identify and screen additional potential studies for inclusion.

### Data Extraction 

Data on primary author, year of publication, country of study origin, setting, study design, sample size, comparison group, sample age, exposure assessment, outcome definition, outcome ascertainment, covariates, subgroups, and results were abstracted from included studies. Two of us (A.B. and S.C.) independently assessed the risk of bias in the included studies using the Newcastle-Ottawa Scale (score range: 0-9 for cohort and case-control studies and 0-10 for cross-sectional studies, with higher scores indicating higher-quality studies).^34^

We compared study authors, start and end dates, and data sources to identify overlapping study samples. In circumstances in which multiple studies reported outcomes on the same study sample, the study that was most relevant to the objectives of the review was included. For studies that reported separate results for self-injurious behavior and suicidality, both results were included for analysis.

To perform subgroup analyses by self-harm type, we combined studies with outcomes that were associated with suicidal ideation, suicide attempt, or suicide under the term suicidality. We defined self-injurious behavior as nonaccidental behavior that resulted in self-inflicted physical injury but had no specified intent of suicide or sexual arousal.^35^ We stratified results according to self-harm type (ie, self-injurious behavior or suicidality), and we pooled the results given that both types of self-harm co-occur at high rates in both children and adults^27,36,37,38^ and may share important correlates, such as depression, compensatory regulation, and substance use disorder.^39,40^ Studies that included children (aged <20 years) and adults (aged ≥20 years) were included in both subgroup analyses if relevant outcomes for each age group were reported separately. When adult and pediatric age groups were not reported separately, the study was categorized as pediatric if the mean age of participants at enrollment was younger than 20 years, and the study was categorized as adult if the mean age of participants at enrollment was 20 years or older. Study setting was defined as clinical if participants were evaluated in clinical settings, such as outpatient clinics or emergency departments, or nonclinical if participants were recruited through community settings, registries, or databases.

### Statistical Analysis

Pooled ORs and 95% CIs were estimated using the DerSimonian-Laird random-effects model.^41^ A random-effects model was chosen a priori to allow for heterogeneity across studies and generalization of findings. Meta-regressions were performed with potential moderators, including age group, setting, quality score, sample size, year of publication, sex, presence or absence of comorbid conditions (including other neuropsychiatric conditions, such as attention-deficit/hyperactivity disorder and intellectual disability), and continent when at least 4 studies provided relevant data. Heterogeneity was assessed using *I*^2^ tests and the Cochran *Q* test^42,43^ based on γ distribution as proposed by Kulinskaya and Dollinger.^44^ In addition, 95% prediction intervals were computed to estimate the variation of the true effect size (ie, the distribution of the effect sizes in comparable populations). Publication bias was assessed using the Egger and Tang tests, sample size–based funnel plots,^45^ and trim-and-fill funnel plots by imputing missing studies that would be needed to eliminate publication bias (ie, symmetric and inverted funnel plot) based on the L_0_ estimator. Summary ORs and 95% CIs were recomputed according to these imputed studies. Analyses were performed using the *meta* package in R and Stata, version 16 (StataCorp LLC).

## Results

**Figure 1.**
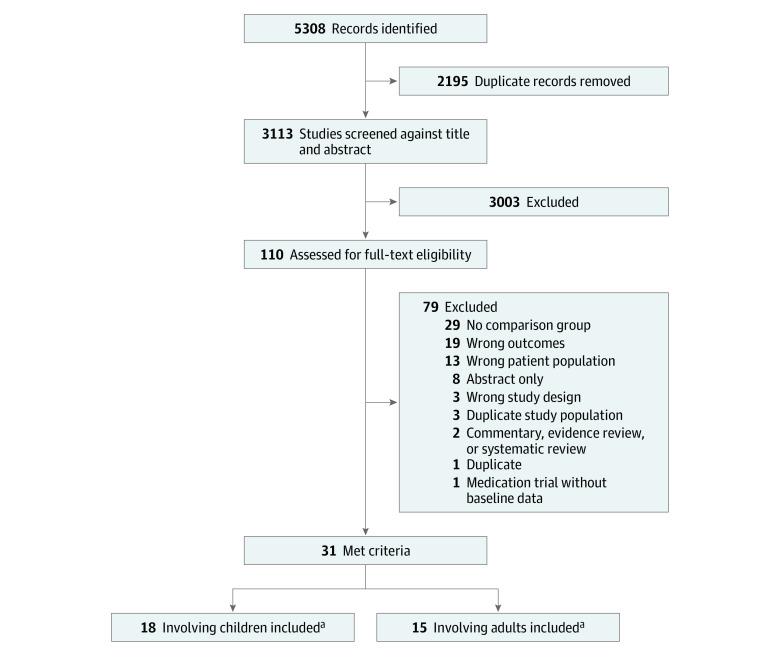
Study Selection ^a^Two studies involved both adults and children.

The initial database search identified 5304 records, and Embase search alerts subsequently identified 4 additional studies, resulting in a total of 5308 records (Figure 1). After removing 2195 duplicates, we screened 3113 titles and abstracts, of which 3003 were excluded during title and abstract review. After a full-text review of 110 potentially eligible studies, we excluded 79 for reasons such as lack of comparison group, wrong outcomes, or wrong or duplicate patient population, leaving 31 studies that met the inclusion criteria.

A total of 36 results were retrieved from the 31 studies.^9,16,17,18,46,47,48,49,50,51,52,53,54,55,56,57,58,59,60,61,62,63,64,65,66,67,68,69,70,71,72^ Twenty-nine of the 36 results showed a significant association between ASD and self-harm,^9,18,48,49,50,51,52,53,54,55,56,57,58,60,61,63,64,66,67,68,69,70,71,72^ and people with ASD were at similarly increased risk of both self-injurious behavior and suicidality. Included studies were published from 1999 to 2021. Of the 5 studies with multiple observed results, 2 reported results separately for pediatric and adult populations^49,64^ and 3 reported results for separate relevant outcomes.^48,60,72^

### Study Characteristics

The 31 studies were heterogeneous in the comparison group chosen, ASD ascertainment, age groups included, and study setting (Table 1). Overall, 31 studies from 11 countries were identified, including 14 studies (45%) from Europe,^17,46,47,48,50,52,55,57,60,61,64,67,68,69^ 13 studies (42%) from North America,^9,16,51,53,54,56,58,59,63,65,66,70,72^ and 4 studies (13%) from Asia.^18,49,62,71^

**Table 1.** Characteristics of the Studies Included in the Meta-analysis

Source (country of study origin)	Sample age, y	Age group	Setting	Comparison group	Self-harm outcome	Sample size, No.	Controls for covariates?	Study design	Quality score^a^
Agnafors et al,^46^ 2020 (Sweden)	0-17	Pediatric	Nonclinical, database	Participants without ASD or developmental delays	Self-injurious behavior	359 597	Yes	Cross-sectional	10/10
Buono et al,^47^ 2010 (Italy)	1-47	Pediatric	Nonclinical	Participants without ID or ASD	Self-injurious behavior	84	No	Cross-sectional	4/10
Cassidy et al,^48^ 2018 (England)	20-60	Adult	Nonclinical	Participants without ASD	Self-injurious behavior and suicidality	333	No	Cross-sectional	7/10
Chen et al,^49^ 2017 (Taiwan)	12-29	Pediatric and adult	Nonclinical, database	Age- and sex-matched control participants without ASD diagnosis	Suicide attempt	28 090	Yes	Cohort	9/9
Cooper et al,^50^ 2009 (Scotland)	≥16	Adult	Nonclinical	Participants without ASD	Self-injurious behavior	1023	No	Cohort	7/9
Croen et al,^51^ 2015 (United States)	≥18	Adult	Nonclinical	Participants without ASD	Suicide attempt	16 577	No	Cohort	7/9
Culpin et al,^17^ 2018 (England)	7-16	Pediatric	Nonclinical	Participants without ASD	Self-injurious behavior	2720	Yes	Cohort	9/9
Dell’Osso et al,^52^ 2019 (Italy)	Mean age: 25.7	Adult	Nonclinical	Participants without ASD	Suicidal ideation and suicide attempt	194	No	Cross-sectional	6/10
Dickerson Mayes et al,^53^ 2015 (United States)	6-18	Pediatric	Clinical	Participants without neurodevelopmental disorders or prescribed psychotropic medications	Suicidal ideation and suicide attempt	515	No	Cross-sectional	6/10
Dominick et al,^54^ 2007 (United States)	4-14	Pediatric	Nonclinical	Participants with a history of language impairment	Self-injurious behavior	92	No	Cross-sectional	7/10
Eden et al,^55^ 2014 (United Kingdom)	4-15	Pediatric	Nonclinical	Participants with Down syndrome	Self-injurious behavior	231	No	Cross-sectional	5/10
Fodstad et al,^56^ 2012 (United States)	12-39 mo	Pediatric	Nonclinical	Participants without ASD	Self-injurious behavior	624	No	Cross-sectional	7/10
Folch et al,^57^ 2018 (Spain)	18-84	Adult	Nonclinical	Participants with ID and without ASD	Self-injurious behavior	833	No	Cross-sectional	7/10
Hand et al,^58^ 2020 (United States)	≥65	Adult	Nonclinical, database	Age- and sex-matched control participants without ASD	Self-injurious behavior and suicidality	51 535	Yes	Cross-sectional	10/10
Hardan and Sahl,^59^ 1999 (United States)	4-18	Pediatric	Clinical	Participants without ASD	Suicide ideation	193	No	Cohort	6/9
Hirvikoski et al,^60^ 2020 (Sweden)	0-100 (mean age: 22)	Adult	Nonclinical, registry	Age- and sex-matched control participants without ASD, ID, or ADHD	Suicide and suicide attempt	325 008	Yes	Case-control	9/9
Jokiranta-Olkoniemi et al,^61^ 2021 (Finland)	2-28	Pediatric	Nonclinical, registry	Participants without ASD or severe/profound ID	Self-injurious behavior and suicidality	23 145	Yes	Cohort	7/9
Kalb et al,^9^ 2016 (United States)	3-17	Pediatric	Clinical	Participants without ASD or ID	Self-injurious behavior	6 412 727	Yes	Cross-sectional	8/10
Kamio,^62^ 2002 (Japan)	“Children and adolescents”	Pediatric	Nonclinical	Participants without ASD	Self-injurious behavior	657	No	Cross-sectional	6/10
Kats et al,^63^ 2013 (United States)	30-59	Adult	Nonclinical, database	Participants with ID and without ASD	Self-injurious behavior	4989	No	Cross-sectional	8/10
Kirby et al,^16^ 2019 (United States)	14-70	Adult	Nonclinical, registry	Participants without ASD	Suicide	9 121 537	No	Cohort	7/9
Kõlves et al,^64^ 2021 (Denmark)	≥10	Pediatric and adult	Nonclinical, registry	Participants without ASD	Suicide attempt	6 559 266	Yes	Cohort	8/9
MaClean et al,^65^ 2010 (United States)	18-72 mo	Pediatric	Clinical	Participants without ASD	Self-injurious behavior	196	No	Cross-sectional	6/10
Moses,^66^ 2018 (United States)	13-18	Pediatric	Nonclinical	Participants without ASD	Suicide attempt	10 489	No	Cross-sectional	5/10
Nicholls et al,^67^ 2020 (United Kingdom)	3-19	Pediatric	Nonclinical	Participants in special education without ASD	Self-injurious behavior	321	No	Cross-sectional	6/10
Pelton et al,^68^ 2020 (United Kingdom)	18-90	Adult	Nonclinical	Participants without ASD	Suicidal ideation	689	No	Cross-sectional	7/10
Richards et al,^69^ 2012 (England)	4-62 (mean age: 13)	Pediatric	Nonclinical	Participants with Down syndrome	Self-injurious behavior	197	No	Cross-sectional	7/10
Soke et al,^70^ 2018 (United States)	30-68 mo	Pediatric	Nonclinical	Participants with developmental delay	Self-injurious behavior	1668	Yes	Case-control	8/9
Takara and Kondo,^71^ 2014 (Japan)	18-87	Adult	Clinical	Participants without ASD and with depression	Suicide attempt	336	No	Case-control	7/9
Tani et al,^18^ 2012 (Japan)	18-73	Adult	Nonclinical	ASD vs patients with no mental disorders but with disturbance of social functions and communication skills	Self-injurious behavior	162	No	Cross-sectional	6/10
Vohra et al,^72^ 2016 (United States)	22-64	Adult	Clinical	Participants without ASD	Self-injurious behavior and suicidality	102 108	No	Cross-sectional	8/10

Abbreviations: ADHD, attention-deficit/hyperactivity disorder; ASD, autism spectrum disorder; ID, intellectual disability.

^a^ The Newcastle-Ottawa Scale for quality assessment can award a maximum score of 10 for cross-sectional studies and a maximum score of 9 for case-control and cohort studies. A score range of 7 to 10 indicates high quality, 5 to 6 indicates moderate quality, and 0 to 4 indicates low quality in cross-sectional studies. A score range of 6 to 9 indicates high quality, 4 to 5 indicates moderate quality, and 0-4 indicates low quality in cohort and case control studies.

The study populations spanned the entire age spectrum from toddlers to older adults. Six studies (19%) combined pediatric and adult populations,^16,47,49,60,64,69^ of which 2 studies reported relevant analyses by age group.^49,64^ Of the remaining 4 studies, 2 studies were categorized as having a pediatric population, with a mean participant age of younger than 20 years at the time of enrollment,^47,69^ and 2 studies were categorized as having an adult population with a mean participant age of 20 years or older at the time of enrollment.^16,60^ The final subgroup analysis by age group included 16 studies (52%) in pediatric populations,^9,17,46,47,53,54,55,56,59,61,62,65,66,67,69,70^ 13 studies (42%) in adult populations,^16,18,48,50,51,52,57,58,60,63,68,71,72^ and 2 studies (6%) in both pediatric and adult populations^49,64^ (Table 1).

The study quality was, overall, moderate to high, with higher numbers representing a lower degree of potential bias. Case-control studies were assigned a mean score of 8 of 9, cohort studies were assigned a mean score of 7.5 of 10, and cross-sectional studies were assigned a mean score of 6.8 of 10 (Table 1).

Of the 31 studies, 6 (19%) recruited participants in a clinical setting^9,53,59,65,71,72^ and 25 (81%) recruited participants in a nonclinical setting^16,17,18,46,47,48,49,50,51,52,54,55,56,57,58,60,61,62,63,64,66,67,68,69,70^ (eFigure 1 in the Supplement). Most studies (n = 22 [71%]) used the general population without ASD as the control group.^9,16,17,46,47,48,49,50,51,52,53,56,58,59,60,61,62,64,65,66,68,72^ Nine studies (29%) used individuals with neurodevelopmental or neuropsychiatric conditions or special education eligibility without ASD as control participants.^18,54,55,57,63,67,69,70,71^ Fifteen studies (48%) investigated self-injurious behavior without suicidality,^9,18,46,47,50,54,55,56,57,62,63,65,67,69,70^ 14 studies (45%) investigated suicidality (ie, suicidal ideation, suicide attempt, or suicide),^16,17,49,51,52,53,58,59,60,61,64,66,68,71^ and 2 studies (6%) investigated both.^48,72^

### Summary of Findings

**Figure 2.**
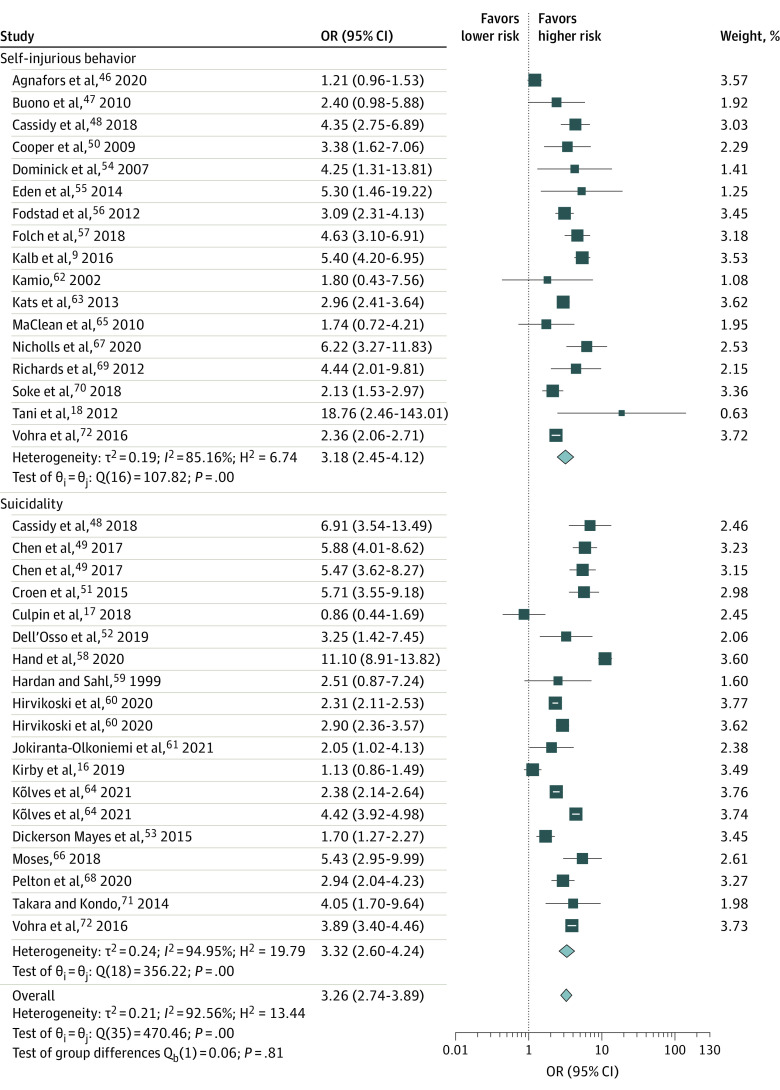
Forest Plot of the Association of Autism Spectrum Disorder (ASD) and Risk of Self-injurious Behavior, Suicidality, and Overall Self-harm The length of the horizontal lines represents the 95% CIs. The size of the squares represents the weighted odds ratios (ORs), and the size of the diamonds represents summary ORs.

Of the 36 results retrieved from 31 studies, 29 revealed significant associations between ASD and self-harm, and 7 showed no significant association. In pooled data, people with ASD had 2.26-times higher odds of self-harm than those without ASD (pooled OR, 3.26; 95% CI, 2.74-3.89; *I*^2^ = 92.56%). Seventeen studies^9,18,46,47,48,50,54,55,56,57,62,63,65,67,69,70,72^ assessed the association between ASD and self-injurious behavior and reported ORs that ranged from 1.21 to 18.76. Sixteen studies^16,17,48,49,51,52,53,58,59,60,61,64,66,68,71,72^ assessed the association between ASD and suicidality and reported ORs that ranged from 0.86 to 11.10. Individuals with ASD were at similarly heightened risk of self-injurious behavior (pooled OR, 3.18; 95% CI, 2.45-4.12; *I*^2^ = 85.16%) and suicidality (pooled OR, 3.32; 95% CI, 2.60-4.24; *I*^2^ = 94.95%) (Table 2; Figure 2).

**Table 2.** Summary Estimates of ORs and 95% CIs of Self-harm Associated With Autism Spectrum Disorder by Study Characteristics

Study characteristic	OR (95% CI)		
	Self-injurious behavior	Suicidality	Self-harm (self-injurious behavior or suicidality)
Overall	3.18 (2.45-4.12)	3.32 (2.60-4.24)	3.26 (2.74-3.89)
Children	2.99 (1.93-4.64)	2.53 (1.70-3.76)	2.74 (2.17-3.44)
Adults	3.38 (2.54-4.50)	3.84 (2.78-5.30)	3.97 (3.11-5.01)
Setting			
Clinical	NA	NA	2.93 (2.07-4.16)
Nonclinical	NA	NA	3.37 (2.73-4.16)
Asia	NA	NA	5.38 (4.05-7.14)
Europe	NA	NA	2.98 (2.40-3.72)
North America	NA	NA	3.22 (2.32-4.47)

Abbreviations: NA, not available; OR, odds ratio.

**Figure 3.**
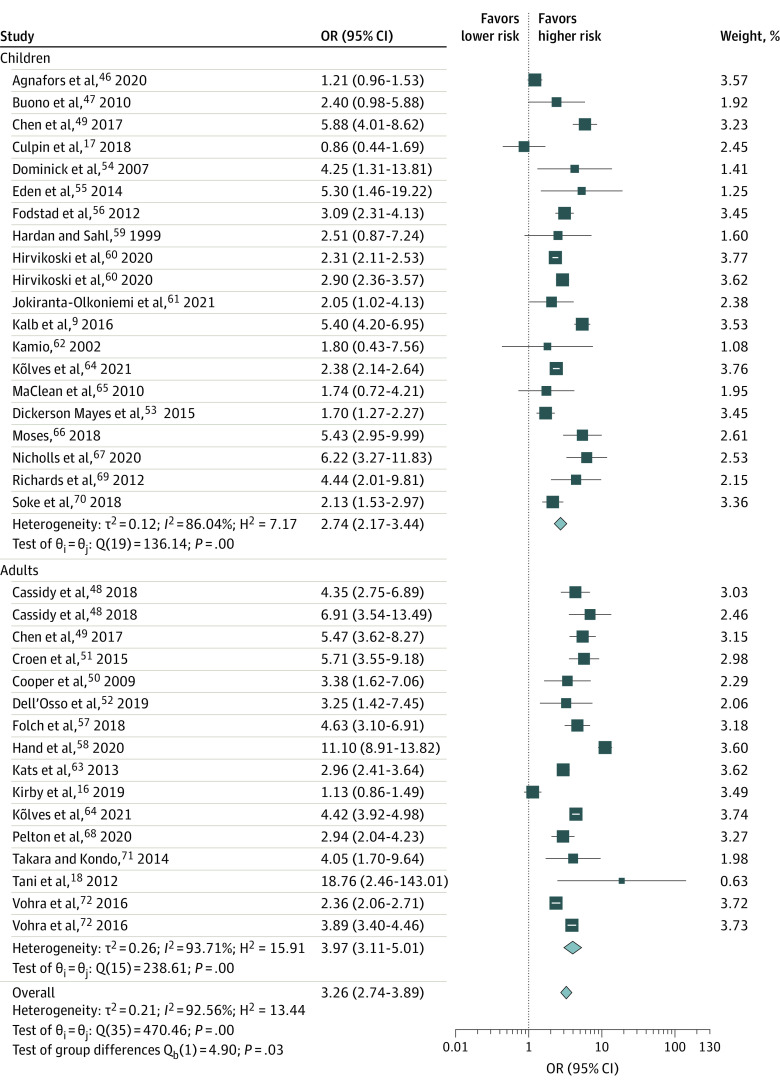
Forest Plot of the Association of Autism Spectrum Disorder (ASD) and Risk of Self-injurious Behavior and Suicidality Among Children and Adults The length of the horizontal lines represents the 95% CIs. The size of the squares represents the weighted odds ratios (ORs), and the size of the diamonds represents summary ORs.

In meta-regression analyses, only age group significantly moderated the association between ASD and self-harm, where adults were at greater risk of self-harm than children (OR, 1.45; 95% CI, 1.04-2.03). The pooled ORs for both self-injurious behavior and suicidality were similarly high in adults (pooled self-harm OR, 3.97 [95% CI, 3.11-5.01; *I*^2^ = 93.71%]; self-injurious behavior OR, 3.38 [95% CI, 2.54-4.50; *I*^2^ = 74.12%]; and suicidality OR, 3.84 [95% CI, 2.78-5.30; *I*^2^ = 96.09%]) and in children (pooled self-harm OR, 2.74 [95% CI, 2.17-3.44; *I*^2^ = 86.04%]; self-injurious behavior OR, 2.99 [95% CI, 1.93-4.64; *I*^2^ = 88.66%]; and suicidality OR, 2.53 [95% CI, 1.70-3.76; *I*^2^ = 85.89%]) (Table 2; Figure 3). Results were consistent in the direction and magnitude of the association across continents (eFigure 2 in the Supplement). We were unable to consider sex and comorbid conditions as potential moderators because the studies did not provide enough information about these factors to allow appropriate analyses.

The trim-and-fill funnel plots did not indicate any major publication bias in any of the meta-analyses. Pooled ORs based on combined observed and imputed studies still showed significant associations between ASD and self-harm in the pediatric and adult populations (eFigures 3-5 in the Supplement). When 6 studies were imputed to the meta-analysis, the overall OR decreased from 3.26 (based on 36 observed results from 31 studies) to 2.82 (based on 42 observed and imputed results) (eFigure 3 in the Supplement). In addition, both the Egger test and Tang test showed nonsignificant publication bias.

Effect size estimates indicated a high level of heterogeneity across studies in all sets of meta-analyses, with the Cochrane *Q* statistic ranging from 19.32 (*df* = 5; *P* < .001) to 469.36 (*df* = 35; *P* < .001) for the pooled meta-analysis. The estimated mean E_th_ [Q] of the null distribution of Q was 21.15, and the corrected mean E[Q] was 21.42 with an estimated variance of 40.06. The parameters of the approximating γ distribution were α = 11.45 and β = 1.87, with a *P* < .001. The Breslow-Day statistic value was 231.65 (*P* < .001). Despite a high level of heterogeneity, the results showed consistent significant associations between ASD and self-harm, with all but 1 study indicating an increased odds of self-harm among people with ASD.

## Discussion

In the US, the incidence of suicide attempts or suicidal ideation in children aged 5 to 19 years who are treated in emergency departments has doubled in the past decade,^73^ and recent studies suggest that people with ASD are at a particularly heightened risk of self-harm.^24,74,75^ The present meta-analysis found that ASD was associated with more than a 3-fold increase in odds of self-injurious behavior and suicidality. These findings were generally consistent across children and adults and across geographic regions. The substantially increased odds of self-harm associated with ASD was robust regardless of study designs and methods, settings, and populations. The 3 funnel plots visually demonstrated appropriate symmetry and scatter, indicating limited publication bias, and imputation of missing studies did not substantially change the estimated ORs. Because heightened odds of self-harm were observed in both children and adults, targeted interventions to identify and mitigate the risk are imperative.

The findings of this study are of public health importance given the continuing increase in ASD prevalence and the high prevalence of self-injurious behavior in individuals with ASD. A recent meta-analysis investigated self-injurious behavior among people with ASD and reported a pooled prevalence estimate of 42% (95% CI, 38%-47%), but it did not investigate the comparative risk or the risk of suicidality.^23^ We included 31 studies with samples that cover a wide range of ages and self-harm outcomes, creating substantial heterogeneity. Although the *I*^2^ statistic showed the proportion of the variance in observed outcomes that reflected variation in true effect sizes rather than a sampling error, it did not measure the absolute variance of true effect sizes.^76^ It is the proportion of total variation in the point estimates that is attributable to between-study heterogeneity.^77^ In contrast, the prediction interval shows the range of the absolute amount of dispersion in true effect sizes.^76^ In this study, the true effect size (ie, OR for association of ASD and self-harm) likely is between 1.26 and 8.47.

### Limitations

This study has several limitations. First, the estimates of the association between ASD and self-harm were not adjusted for comorbidities, such as attention-deficit/hyperactivity disorder and intellectual disability. Although the role of comorbid conditions warrants further investigation, comorbidities are unlikely to fully explain the substantially increased odds of self-harm among people with ASD.^64^ Second, we found that substantial heterogeneity among the observational studies in this systematic review can be partly explained by the differences in study age groups, definitions, designs, analytic approaches, and comorbidities that were infrequently considered as covariates. Third, the outcomes of self-injurious behavior and suicidality varied broadly in their clinical presentations, patient and family burden, and interventions. This study synthesized the epidemiologic evidence for the 2 types of self-harm but did not offer estimates for each specific type of self-harm. Fourth, as in most studies that explore epidemiologic patterns in people with ASD, the linear positive cohort effects that demonstrate year-to-year increases in autism diagnoses are important to note.^78^ In this study, the cohort effects may introduce bias that is associated with misclassification of ASD diagnosis in older populations, possibly underestimating the risk of self-harm in adults with ASD. Most studies included in this meta-analysis involved children, although adults represent a larger proportion of the population with autism.^79^ This situation reflects a greater awareness and more appropriate diagnosis of ASD in the past few decades that has focused on pediatric practitioners and populations, accompanied with limited research on autism in adulthood. Fifth, we did not exclude studies on the basis of assessment of their risk of bias. Most included studies received Newcastle-Ottawa Scale scores that were reflective of minimal risk of bias, but a few studies received lower scores (4-5 points).

## Conclusions

This systematic review with meta-analysis found that ASD was associated with a substantially increased risk of self-injurious behaviors and suicidality. This finding was consistent in pediatric and adult populations across geographic regions and in study designs, methods, and settings. Further research is needed to examine the role of primary care screenings, preventive mental health services, and lethal means counseling in reducing self-harm among people with ASD.

## Notes

Supplement. **eFigure 1.** Forest Plot Showing the Association of Autism Spectrum Disorder (ASD) and Self-Harm by Study Setting **eFigure 2.** Forest Plot Showing the Association of Autism Spectrum Disorder (ASD) and Self-Harm by Continent **eFigure 3.** Funnel Plot for Self-Harm Associated With Autism Spectrum Disorder (ASD) Among Children and Adults **eFigure 4.** Funnel Plot for Self-Harm Associated With Autism Spectrum Disorder (ASD) Among Adults **eFigure 5.** Funnel Plot for Self-Harm Associated With Autism Spectrum Disorder (ASD) Among Children **eMethods 1.** Deviations from PROSPERO Protocol **eMethods 2.** Search Strategies
