# Supplementary material for: Risk of Self-harm in Children and Adults With Autism Spectrum Disorder: A Systematic Review and Meta-analysis
Source: JAMA Netw Open. 2021 Oct 19;4(10):e2130272. doi: 10.1001/jamanetworkopen.2021.30272 (PMC8527356; doi:10.1001/jamanetworkopen.2021.30272)
Supplement: Supplement. — eFigure 1. Forest Plot Showing the Association of Autism Spectrum Disorder (ASD) and Self-Harm by Study Setting eFigure 2. Forest Plot Showing the Association of Autism Spectrum Disorder (ASD) and Self-Harm by Continent eFigure 3. Funnel Plot for Self-Harm Associated With Autism Spectrum Disorder (ASD) Among Children and Adults eFigure 4. Funnel Plot for Self-Harm Associated With Autism Spectrum Disorder (ASD) Among Adults eFigure 5. Funnel Plot for Self-Harm Associated With Autism Spectrum Disorder (ASD) Among Children eMethods 1. Deviations from PROSPERO Protocol eMethods 2. Search Strategies [file jamanetwopen-e2130272-s001.pdf]

Supplementary Online Content

Blanchard A, Chihuri S, DiGuiseppi CG, Li G. Risk of self-harm in children and adults with autism spectrum disorder: a systematic review and meta-analysis. *JAMA Netw Open*. 2021;4(10):e2130272. doi:10.1001/jamanetworkopen.2021.30272

eFigure 1. Forest Plot Showing the Association of Autism Spectrum Disorder (ASD) and Self-Harm by Study Setting

eFigure 2. Forest Plot Showing the Association of Autism Spectrum Disorder (ASD) and Self-Harm by Continent

eFigure 3. Funnel Plot for Self-Harm Associated With Autism Spectrum Disorder (ASD) Among Children and Adults

eFigure 4. Funnel Plot for Self-Harm Associated With Autism Spectrum Disorder (ASD) Among Adults

eFigure 5. Funnel Plot for Self-Harm Associated With Autism Spectrum Disorder (ASD) Among Children

eMethods 1. Deviations from PROSPERO Protocol

eMethods 2. Search Strategies

This supplementary material has been provided by the authors to give readers additional information about their work.

© 2021 Blanchard A et al. *JAMA Network Open.*

eFigure 1. Forest Plot Showing the Association of Autism Spectrum Disorder (ASD) and Self-Harm by Study Setting
*The subscripts a (self-injurious behavior) and b (suicidality) for Cassidy et al[40]; the subscripts a (pediatric) and b (adult) for Chen et al[41]; the subscripts a (comparing participants with autism spectrum disorder and without intellectual disability or attention deficit hyperactivity disorder to controls) and b (comparing participants with autism spectrum disorder, intellectual disability, and attention deficit hyperactivity disorder, to controls) for Hirvikoski et al[52]; the subscripts a (pediatric) and b (adult) for Kolves et al[56]; and the subscripts a (self-harm) and b (suicidal ideation) for Vohra et al[64] indicate separate populations within the studies.

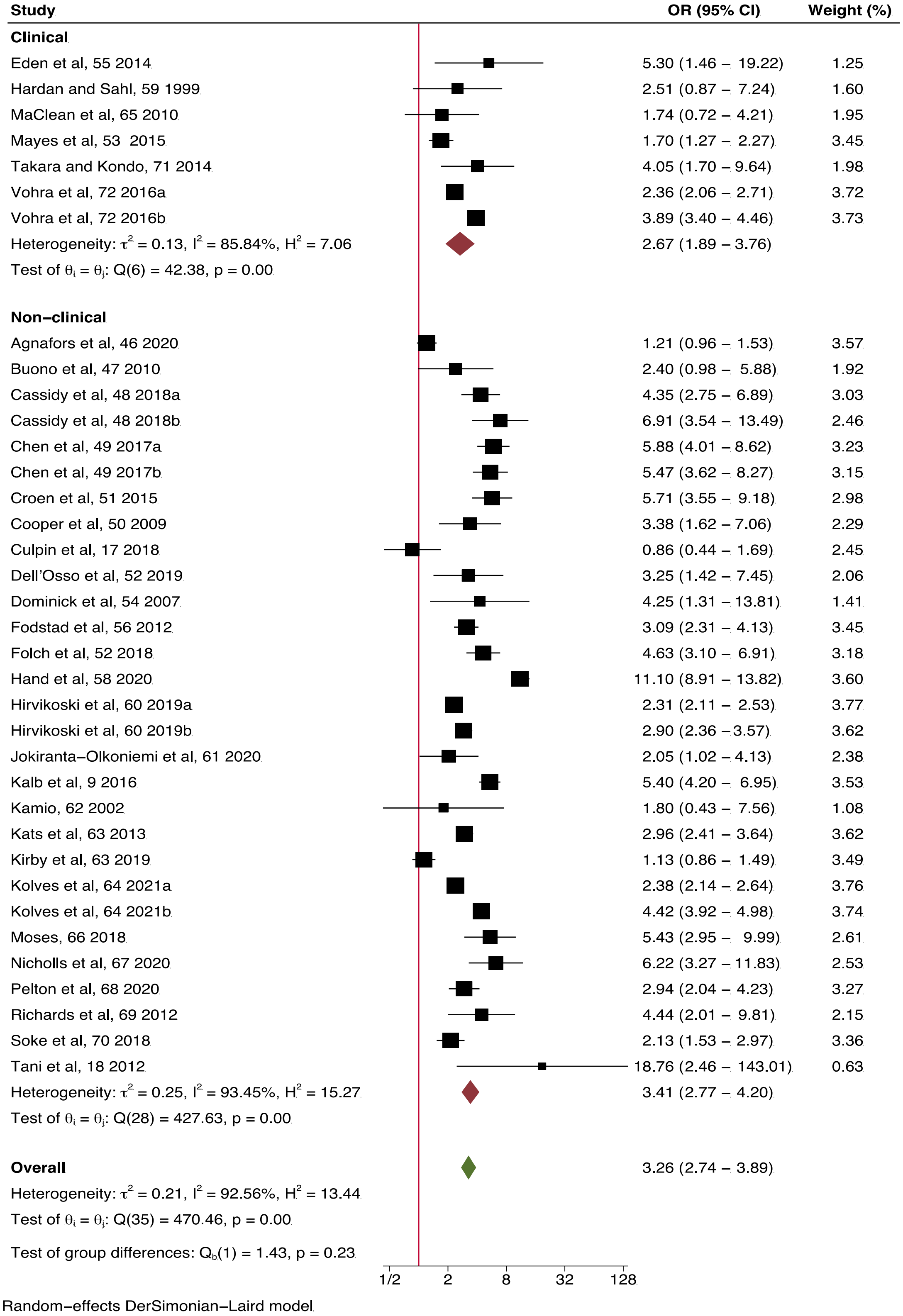

© 2021 Blanchard A et al. *JAMA Network Open.*

eFigure 2. Forest Plot Showing the Association of Autism Spectrum Disorder (ASD) and Self-Harm by Continent
*The subscripts a (self-injurious behavior) and b (suicidality) for Cassidy et al[40]; the subscripts a (pediatric) and b (adult) for Chen et al[41]; the subscripts a (comparing participants with autism spectrum disorder and without intellectual disability or attention deficit hyperactivity disorder to controls) and b (comparing participants with autism spectrum disorder, intellectual disability, and attention deficit hyperactivity disorder, to controls) for Hirvikoski et al[52]; the subscripts a (pediatric) and b (adult) for Kolves et al[56]; and the subscripts a (self-harm) and b (suicidal ideation) for Vohra et al[64] indicate separate populations within the studies.

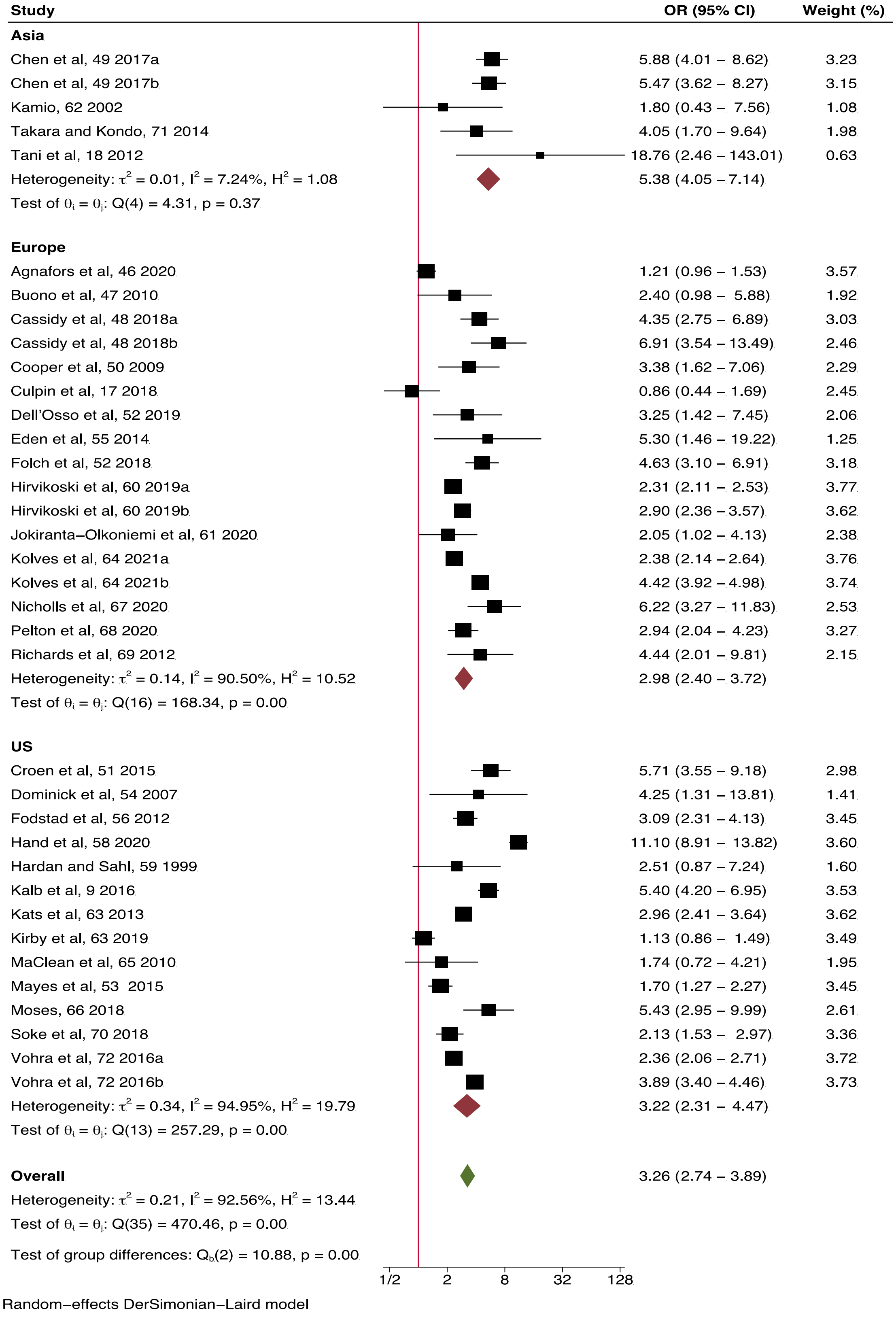

© 2021 Blanchard A et al. *JAMA Network Open.*

eFigure 3. Funnel Plot for Self-Harm Associated With Autism Spectrum Disorder (ASD) Among Children and Adults

When 6 studies are imputed and added to the meta-analysis, the overall odds ratio reduces from 3.26 (based on 36 observed studies) to 2.82 (based on 42 observed and imputed studies).

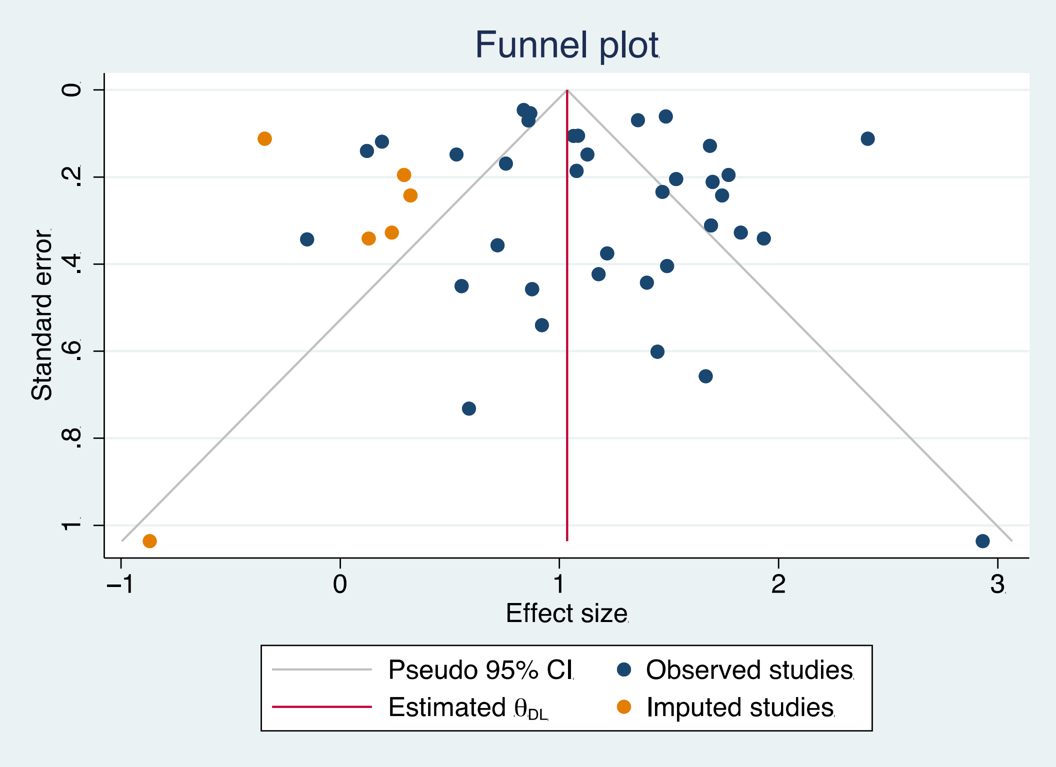

© 2021 Blanchard A et al. *JAMA Network Open.*

eFigure 4. Funnel Plot for Self-Harm Associated With Autism Spectrum Disorder (ASD) Among Adults

When 1 study is imputed and added to the meta-analysis, the overall odds ratio reduces from 3.76 (based on 18 observed studies) to 3.68 (based on 19 observed and imputed studies).

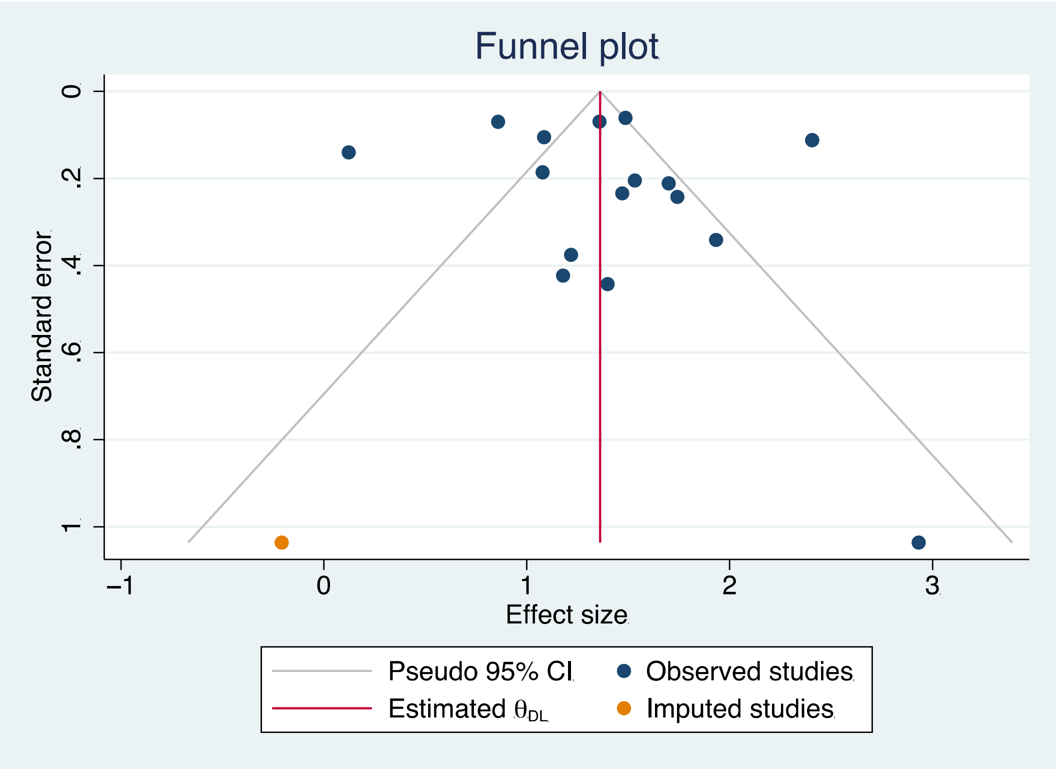

© 2021 Blanchard A et al. *JAMA Network Open.*

eFigure 5. Funnel Plot for Self-Harm Associated With Autism Spectrum Disorder (ASD) Among Children

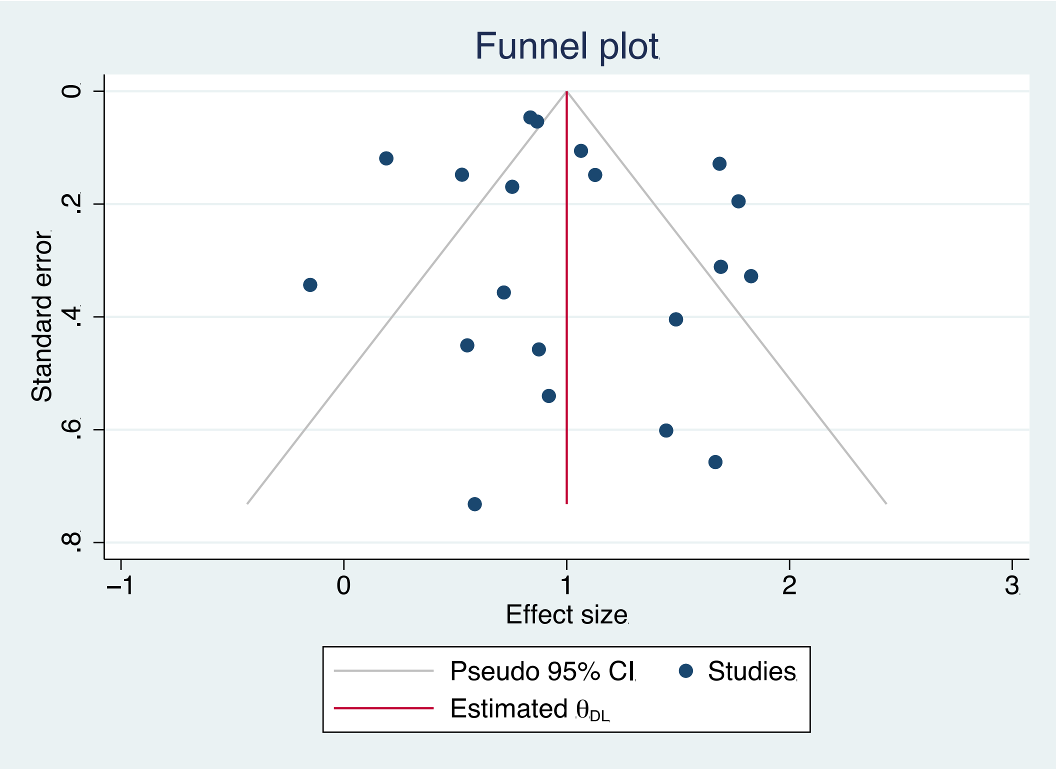

© 2021 Blanchard A et al. *JAMA Network Open.*

eMethods 1. Deviations from PROSPERO Protocol

1) The PROSPERO protocol described an age limit of less than 25 years. We deviated from this protocol to include studies of all age ranges as we found many studies included adults across the age spectrum. We also found the literature search identified a substantial body of evidence describing self-harm in ASD across the life-span, and found this to be valuable information to include.
2) The PROSPERO protocol described excluding studies that collect self-reported data on self-harm for periods greater than the preceding 12 months. After the literature was compiled we found that many high-quality studies reported life-time prevalence of self-harm and decided to include such studies in our analysis.

© 2021 Blanchard A et al. *JAMA Network Open.*

eMethods 2. Search Strategies

PubMed search:
((("asperger syndrome"[MeSH Terms] OR (asperger[tiab] AND syndrome[tiab]) OR "asperger syndrome"[tiab]) OR ("autistic disorder"[MeSH Terms] OR (autistic[tiab] AND disorder[tiab]) OR "autistic disorder"[tiab] OR "autism"[tiab])) OR ("autism spectrum disorder"[MeSH Terms] OR (autism[tiab] AND spectrum[tiab] AND disorder[tiab]) OR "autism spectrum disorder"[tiab])) AND ((("suicide"[MeSH Terms] OR suicide[tiab]) OR ("self-injurious behavior"[MeSH Terms] OR (self-injur*[tiab] AND behavior[tiab]) OR "self-injurious behavior"[tiab] OR (self[tiab] AND harm[tiab]) OR "self harm"[tiab] OR (self[tiab] AND injur*[tiab]) OR self injur*[tiab])))

Embase search:
Query('autism'/exp OR 'asperger syndrome':ti,ab OR (asperger:ti,ab AND syndrome:ti,ab) OR (autistic:ti,ab AND disorder:ti,ab) OR 'autistic disorder':ti,ab OR autism:ti,ab OR (autism:ti,ab AND spectrum:ti,ab AND disorder:ti,ab) OR 'autism spectrum disorder':ti,ab) AND ('automutilation'/exp OR 'suicide'/exp OR suicide:ti,ab OR ('self injur*':ti,ab AND behavior:ti,ab) OR 'self-injurious behavior*':ti,ab OR (self:ti,ab AND harm:ti,ab) OR 'self harm':ti,ab OR (self:ti,ab AND injur*:ti,ab) OR 'self injur*':ti,ab)

PsychInfo Search:
(DE "Self-Injurious Behavior" OR DE "Head Banging" OR DE "Self-Inflicted Wounds" OR DE "Self-Mutilation" OR DE "Self-Poisoning" OR DE "Suicide" OR DE "Attempted Suicide" OR DE "Suicidality" OR (TI "self harm") OR (AB "self harm") OR (TI "self-injurious behavior*") OR (AB "self-injurious behavior*") OR (TI “self injur*”) OR (AB "self injur*") OR (TI “self" AND "injur*”) OR (AB "self" AND "injur*") OR (TI suicide) OR (AB suicide)) AND ((DE "Autism Spectrum Disorders") OR (TI “Asperger syndrome”) OR (AB “Asperger syndrome”) OR (TI “autism spectrum disorder”) OR (AB “autism spectrum disorder”) OR (TI “autistic” AND “disorder”) OR (AB “autistic” AND “disorder”) OR (TI “autistic disorder”) OR (AB “autistic disorder”) OR (TI “autism”) OR (AB “autism”) OR (TI “autism” and “spectrum”) OR (AB “autism” and “spectrum”))

Web of Science Search:
((Autism OR ‘asperger syndrome' OR (asperger AND syndrome) OR (autistic AND disorder) OR 'autistic disorder' OR (autism AND spectrum AND disorder) OR 'autism spectrum disorder') AND (automutilation OR suicide OR 'self-injurious behavior* OR (self AND harm) OR 'self harm' OR (self AND injur*:) OR 'self injur*'))

CINAHL Search:
((MH "Asperger Syndrome") OR (MH "Autistic Disorder") OR (TI “Asperger syndrome”) OR (AB “Asperger syndrome”) OR (TI “autism spectrum disorder”) OR (AB “autism spectrum disorder”) OR (TI “autistic” AND “disorder”) OR (AB “autistic” AND “disorder”) OR (TI “autistic disorder”) OR (AB “autistic disorder”) OR (TI “autism”) OR (AB “autism”) OR (TI “autism” and “spectrum”) OR (AB “autism” and “spectrum”)) AND (MH "Injuries, Self-Inflicted") OR (MH "Suicide") OR (MH "Suicide, Attempted") OR (MH "Suicidal Ideation") OR (TI "self harm") OR (AB "self harm") OR (TI "self-injurious behavior*") OR (AB "self-injurious behavior*") OR (TI “self injur*”) OR (AB "self injur*") OR (TI “self" AND "injur*”) OR (AB "self" AND "injur*") OR (TI suicide) OR (AB suicide))

© 2021 Blanchard A et al. *JAMA Network Open.*
